# Designing 3-Dimensional *In Vitro* Oviduct Culture Systems to Study Mammalian Fertilization and Embryo Production

**DOI:** 10.1007/s10439-016-1760-x

**Published:** 2016-11-14

**Authors:** Marcia A. M. M. Ferraz, Heiko H. W. Henning, Tom A. E. Stout, Peter L. A. M. Vos, Bart M. Gadella

**Affiliations:** 1 Department of Farm Animal Health, Faculty of Veterinary Medicine, Utrecht University, Yalelaan 104, 3584CM Utrecht, The Netherlands; 2 Department of Equine Sciences, Faculty of Veterinary Medicine, Utrecht University, Yalelaan 112, 3584CM, Utrecht, The Netherlands; 3 Department of Biochemistry and Cell Biology, Faculty of Veterinary Medicine, Utrecht University, Yalelaan 79, 3584CM Utrecht, The Netherlands

**Keywords:** 3-D culture, Microfluidics, Bio-engineering, Fallopian tube, Polarized epithelium, Embryo development

## Abstract

The oviduct was long considered a largely passive conduit for gametes and embryos. However, an increasing number of studies into oviduct physiology have demonstrated that it specifically and significantly influences gamete interaction, fertilization and early embryo development. While oviduct epithelial cell (OEC) function has been examined during maintenance in conventional tissue culture dishes, cells seeded into these two-dimensional (2-D) conditions suffer a rapid loss of differentiated OEC characteristics, such as ciliation and secretory activity. Recently, three-dimensional (3-D) cell culture systems have been developed that make use of cell inserts to create basolateral and apical medium compartments with a confluent epithelial cell layer at the interface. Using such 3-D culture systems, OECs can be triggered to redevelop typical differentiated cell properties and levels of tissue organization can be developed that are not possible in a 2-D culture. 3-D culture systems can be further refined using new micro-engineering techniques (including microfluidics and 3-D printing) which can be used to produce ‘organs-on-chips’, *i.e.* live 3-D cultures that bio-mimic the oviduct. In this review, concepts for designing bio-mimic 3-D oviduct cultures are presented. The increased possibilities and concomitant challenges when trying to more closely investigate oviduct physiology, gamete activation, fertilization and embryo production are discussed.

## The oviduct

**Figure 1:**
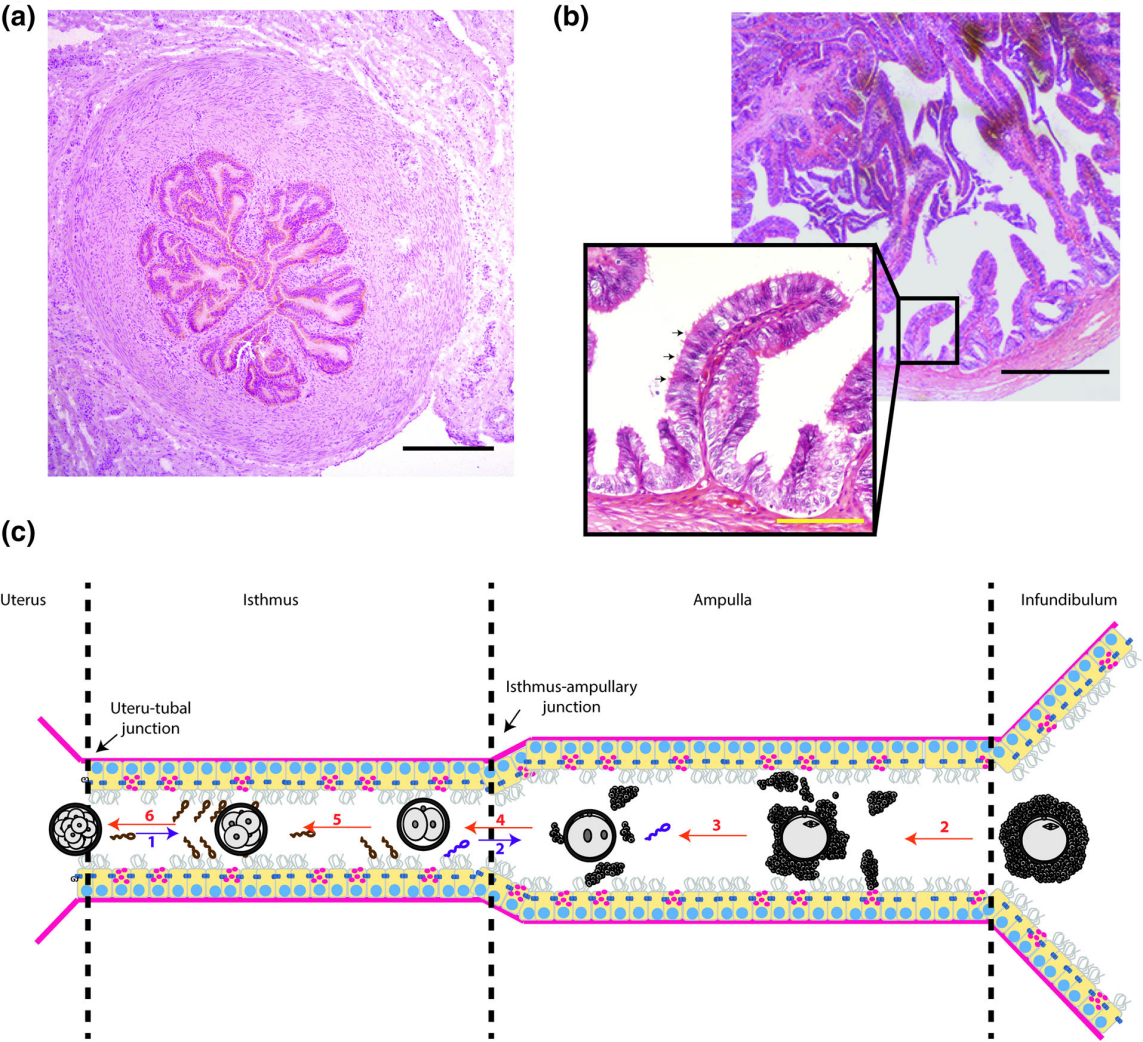
Histological images from bovine oviduct sections of (a) the isthmus (b) the ampulla (with an enlargment of a folded area to show the organization of the cuboid to columnar epithelial cells; black arrows indicate ciliated cells). Note the thicker stroma and muscular wall, and smaller lumen, of the isthmus (a) compared to the ampulla (b), and the higher degree of folding of the ampulla. (c) A schematic view of the entire length of the oviduct including the different segments: the utero-tubal junction, the isthmus, the isthmus-ampullary junction, the ampulla and the infundibulum, respectively. In this schematic view, the various reproductive processes are listed in chronological and spatial order: (1) entry of sperm from the uterine lumen and establishment of a sperm reservoir in the isthmus; (2) at the time of ovulation, the released COC will be captured by the infundibulum, and biochemical changes in the oviduct milieu will stimulate sperm release form the reservoir, and migration to the site of fertilization; (3) the COC will be transported through the ampulla and fertilized by one of the capacitated spermatozoa while, during transport, the COC will gradually lose its cumulus mass; (4) the fertilized oocyte (zygote) will continue its development until the first cell cleavage event; (5) the 2-cell embryo, and after successive cleavages 4, 8 and 16 cell stages are formed (the latter is termed a morula); (6) the morula stage leaves the oviduct via the UTJ and will develop further and implant within the uterus. Black bars = 50* µ*m, yellow bar = 10* µ*m.

The oviduct, or fallopian tube, was first described by Fallopius in 1561 as a presumably passive channel to hold or transport gametes and early embryos in mammals.^75^ The oviduct is a convoluted tube consisting of longitudinal and circular muscular, and a stromal layer lined by a simple cuboidal to columnar epithelium containing both ciliated and secretory cells.^83^,^106^,^123^ The ciliated cells are important for gamete transport and sperm interaction, in particular helping to create a ‘sperm reservoir’; while the secretory cells are responsible for producing oviduct fluid; a mixture of specific cell secretions and serum transudate.^1^,^2^,^10^,^63^,^118^ In adult mammals (including the woman), four anatomical segments can be distinguished along the length of the oviduct; the infundibulum, ampulla, isthmus and utero-tubal junction, respectively^11^,^122^ (Fig. 1c). The fimbriae of the infundibulum are responsible for capturing the cumulus oocyte complex (COC) and ensuring its transport from the ovary into the oviductal tube. The epithelium of the ampulla is highly folded, has the largest diameter of any oviductal segment and is the specific site where fertilization takes place^1^ (Fig. 1b). The ampulla connects to the much narrower isthmic tube (Fig. 1a). Prior to fertilization, sperm entering the oviductal isthmus from the uterus bind to isthmic epithelial cells which help to prolong sperm viability (the formation of a so called “sperm reservoir”).^19^,^96^,^108^,^112^ A limited number of these bound sperm will be released at around the time of ovulation, undergoing the final changes required to achieve fertilizing capacity as they do so, and migrate into the ampullary region^12^ where they will encounter the mature oocyte (Fig. 1c). After fertilization, the developing embryo will migrate along the isthmic tube towards the utero-tubal junction. At the morula (16 cell) stage, the embryo will exit the oviduct and enter the uterine lumen (Fig. 1c), where it will develop further and undergo a series of complicated interactions with the endometrium in preparation for implantation. The oviductal vasculature is composed of branches of the uterine and ovarian arteries and veins, allowing local exchange of metabolites, hormones and signaling molecules between the oviduct, uterus and ovary.^75^

**Figure 2:**
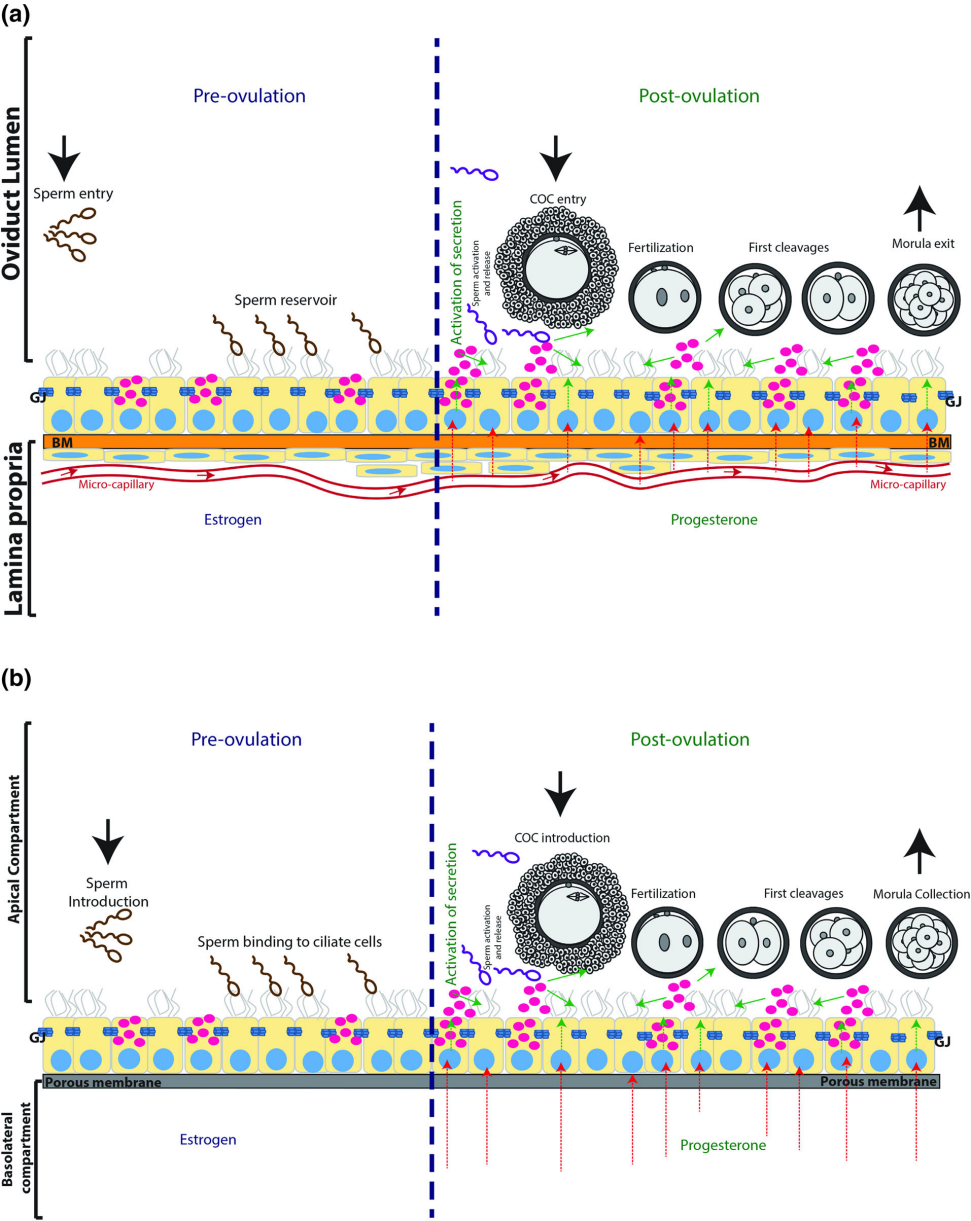
Schematic representation of the oviduct, including its microenvironment before and after ovulation, and of the ideal *in vitro* model of the oviduct. (a): the oviduct epithelium consists of ciliated and non-ciliated (secretory) cells held together in a confluent monolayer of communicating cells by gap junctions (GJ). This epithelium is attached to the luminal side of the basal membrane (BM) which is, in turn, connected to the stroma (containing fibroblast cells and endothelial blood supply) at its peripheral side. Sperm can enter the oviduct and bind to the ciliated cells. This results in the formation of a sperm reservoir during the pre-ovulatory period, under the influence of elevated circulating estrogen concentrations. Ovulation coincides with a switch in endocrine environment in the capillary blood flow of the oviduct. This change stimulates secretory activity in the oviduct epithelium which triggers the release of bound sperm from the isthmus, aids capture of the cumulus-oocyte-complex (COC) and migration of sperm into the ampulla of the oviduct. In the post-ovulatory period, the oviduct is under the influence of progesterone which should promote fertilization and embryo development to the morula stage, when the embryo is ready to leave the oviduct and enter the uterus for further development and implantation. (b) A separation of two compartments with a porous filter, apical reservoir (medium inside the insert) and basolateral reservoir (medium in petri dish), is necessary to mimic the oviduct lumen and lamina propria of the *in vivo* oviduct, respectively (conform (a)). The double perfusion system can be used to simulate peri-ovulatory changes in the blood supply (in the basolateral compartment) and introduce gametes and collect embryos, as would take place in the oviduct *in vivo* (a).

The oviduct is an active organ that orchestrates dynamic changes in its luminal fluid composition to provide optimal microenvironments for gamete maturation/activation, fertilization and early embryo development.^64^ It is the first environment to which an embryo is exposed, and contributes vital factors that affect embryonic development and help atune it to predicted external environmental circumstances during the first 2-6 days post-fertilization, depending on the species^50^,^115^ (Table 1). The successful development of conditions for *in vitro* production (IVP) of embryos for various species has in part been the reason for the relative neglect of the importance of the oviductal microenvironment in early development.^76^ That the oviduct could be successfully by-passed supported the supposition that it was little more than a passive tube for temporarily hosting gametes and embryos.^76^ Nevertheless, it has become clear that not only are fertilization and embryo development less efficient *in vitro* than *in vivo*, but the embryos produced are qualitatively different; a number of studies have now demonstrated the importance of the oviduct for sperm storage and activation,^23^,^32^,^44^,^50^,^53^,^60^,^61^,^73^,^74^,^81^,^91^,^92^,^95^ oocyte modification,^17^,^35^,^38^,^80^ fertilization and early embryo development^6^,^33^,^36^,^38^,^68^,^70^,^79^,^97^,^117^ (Fig. 2a).

**Table 1:** Embryo development within the oviduct of different species (timing is recorded as days after fertilization)

Species	2-cells	4-cells	8-cells	Morula
Woman	1.5 days	2 days	3 days	4 days
Cow	1.5 days	2 days	2.5 days	3.5 days
Sow	0.75 h	1.5 days	2 days	4.5 days
Mare	1 day	1.5 days	3 days	5.5 days
Ewe	1 day	1.5 days	2 days	3 days
Mouse	1.5 days	2 days	2.5 days	3 days

## Studying Oviduct Function

Due to its intra-abdominal location, it is difficult to access the delicate interior of the oviduct for experimental studies in situ. It is possible to ligate and excise the oviduct from experimental animals and given reproductive stages and to fix the tissues for histological or other microscopic investigation.^13^,^40^ It is also possible to harvest epithelial cells from recovered oviducts. Methods to culture these oviduct epithelial cells (OEC) can differ with respect to cell isolation techniques, culture conditions and duration, medium used and supplements included.^115^ The aim of the present review is to describe how 3-D culture systems can be designed and modified such that contained OECs mimic their *in vivo* physiology as closely as possible. In this respect, the OECs should at least have a similar morphological appearance and differentiation characteristics and be connected to neighboring cells by tight junctions to form a confluent epithelial cell monolayer. The OECs should also resemble *in vivo* oviduct epithelial cells with respect to protein expression, ciliary and secretory activity, and responses to physiological stimuli.^115^

**Figure 3:**
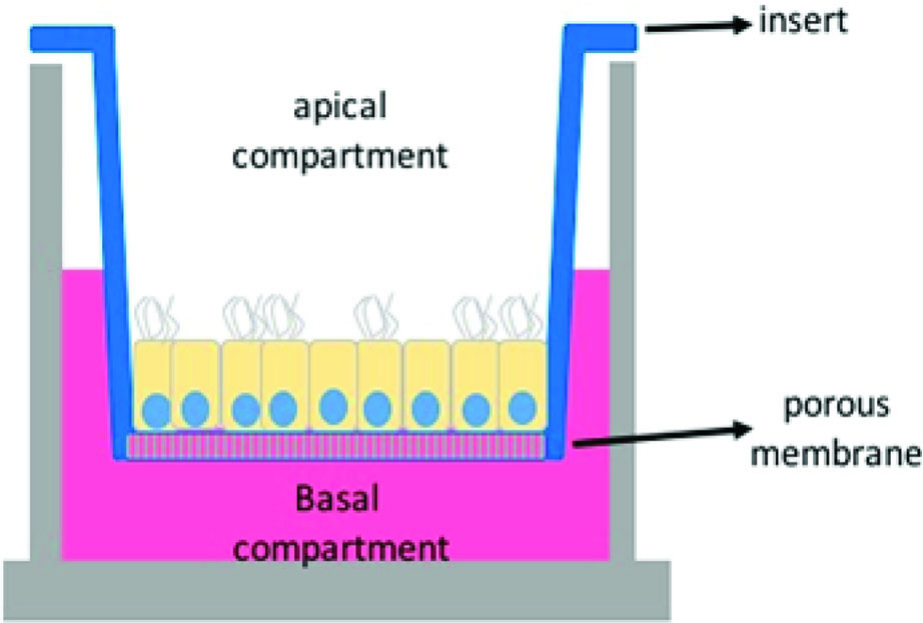
Porous membrane cell culture inserts. In this culture system two compartments are formed (apical and basolateral) that are separated by a porous membrane and a confluent layer of oviduct epithelial cells. This cell culture insert only allows static fluid culture.

An ideal *in vitro* oviduct model should at least allow the possibility to mimic the hormonal changes that occur in the afferent vasculature in the lead up to, and following, ovulation. Moreover, the system should allow the addition and removal of fluids and gametes into the luminal compartment, promote fertilization and allow the culture of embryos to at least the compact morula stage of development (Fig. 2b). These conditions cannot be met when oviduct epithelial cells are simply plated into a petri dish or a cell culture flask. When oviduct epithelial cells are grown in such 2-D cultures they rapidly dedifferentiate into flattened cells without cilia or secretory activity, and also almost completely lose the ability to bind sperm^104^ or to promote fertilization *in vitro*.^72^ Interestingly, with the aid of cell inserts separate compartments (conform Fig. 3) can be created since the medium in the culture dish is separated from the medium in the cell insert, resulting in a basolateral (petri dish) and an apical (cell insert) compartment. OECs can be cultured to confluence on the cell insert and by removing the medium in the insert an air–liquid interface is created that induces the OECs to establish polarity comparable to that seen in situ in the oviduct and to differentiate into active secretory and ciliated cells.^13^,^14^,^34^,^39^,^77^,^90^,^93^

Interestingly, there are no reports of embryo production in these 3-D cell insert-based OEC systems, presumably at least in part because in the insert filters, on which the epithelial cells grow, neither the medium in the petri dish nor that in the cell insert can be perfused to mimic the endocrine changes that will in turn influence OEC function during the peri-ovulatory period. A number of recently introduced technologies may help overcome these short comings: (1) advances in three-dimensional (3-D) printing within biomedical engineering have allowed the creation of scaffolds for live cells, microfluidic devices, and tools for medical imaging.^69^ Since the technology allows rapid printing of prototypes directly from computer-based designs, it is possible to quickly (hours or days) produce novel devices on demand.^69^ The typical folding of the oviduct epithelium (Fig. 1) could be mimicked using these modern 3-D printing approaches. (2) More accurate and miniaturized cell perfusion systems are being developed using microfluidic circuits. When micro-perfusion of both the basolateral (petri dish) and apical (insert) compartments can be achieved, this will mimick the peri-ovulatory hormone changes while simultaneously permitting introduction and collection of gametes and embryos, and sampling of cell secretions. Combining these technologies could result in the creation of a reliable *in vitro* oviduct model to study gamete activation, gamete interaction, fertilization, early embryo development and *in vitro* embryo production. Ultimately, it would be hoped that the embryos produced would be more similar to *in vivo* embryos than IVP embryos produced using current systems. In the following sections, the differences between current OEC culture systems are described in more detail. The type of information that can be gathered from each approach, and their shortcomings, are dealt with. These are further discussed with respect to the anticipated requirements when designing new 3-D culture systems for enhanced gamete maturation, fertilization and early embryo production.

## Approaches to Study Oviduct Function

### *In vivo* and *Ex Vivo*

*In situ* research of oviduct function is difficult due to its intra-abdominal location and tortuous morphology. A single study has reported *in vivo* imaging of pre-labeled sperm cells in the oviduct, using fibered fluorescent confocal microscopy (FCM) in the ewe.^26^ FCM allowed individual spermatozoa to be observed with high resolution in situ in the female genital tract, and moreover to quantitatively track their transit through the uterus and entrance into the oviduct.^26^

Most investigations of oviduct function described as “*in vivo*” are actually *ex vivo* experiments, because the organ was first removed from the animal. These studies are also not entirely *in vitro* because the organ, or at least a part of it, is intact.^116^ Usually, such *ex vivo* intact organ experiments are hampered by a rapid loss of cell viability, which significantly limits the duration of any experiments (several minutes to a few hours). Nevertheless, *ex vivo* organ incubations have been widely used to study sperm migration through the oviduct by video microscopy^57^–^59^,^103^,^107^,^108^ and epifluorescence microscopy.^61^ These techniques are especially applicable to species, like the hamster and the mouse, with a transparent wall to the ampullary region or entire oviduct.^107^However, *ex vivo* approaches are further limited in that they allow only the imaging of physically detectable changes, such as cilia beating and gamete/embryo movement within the oviduct. In addition, the imaging must be done after collecting the oviducts post-mortem or after surgical removal or by using laparoscopy under general anesthesia, all of which are laborious and invasive procedures.

### *In Vitro*

**Figure 4:**
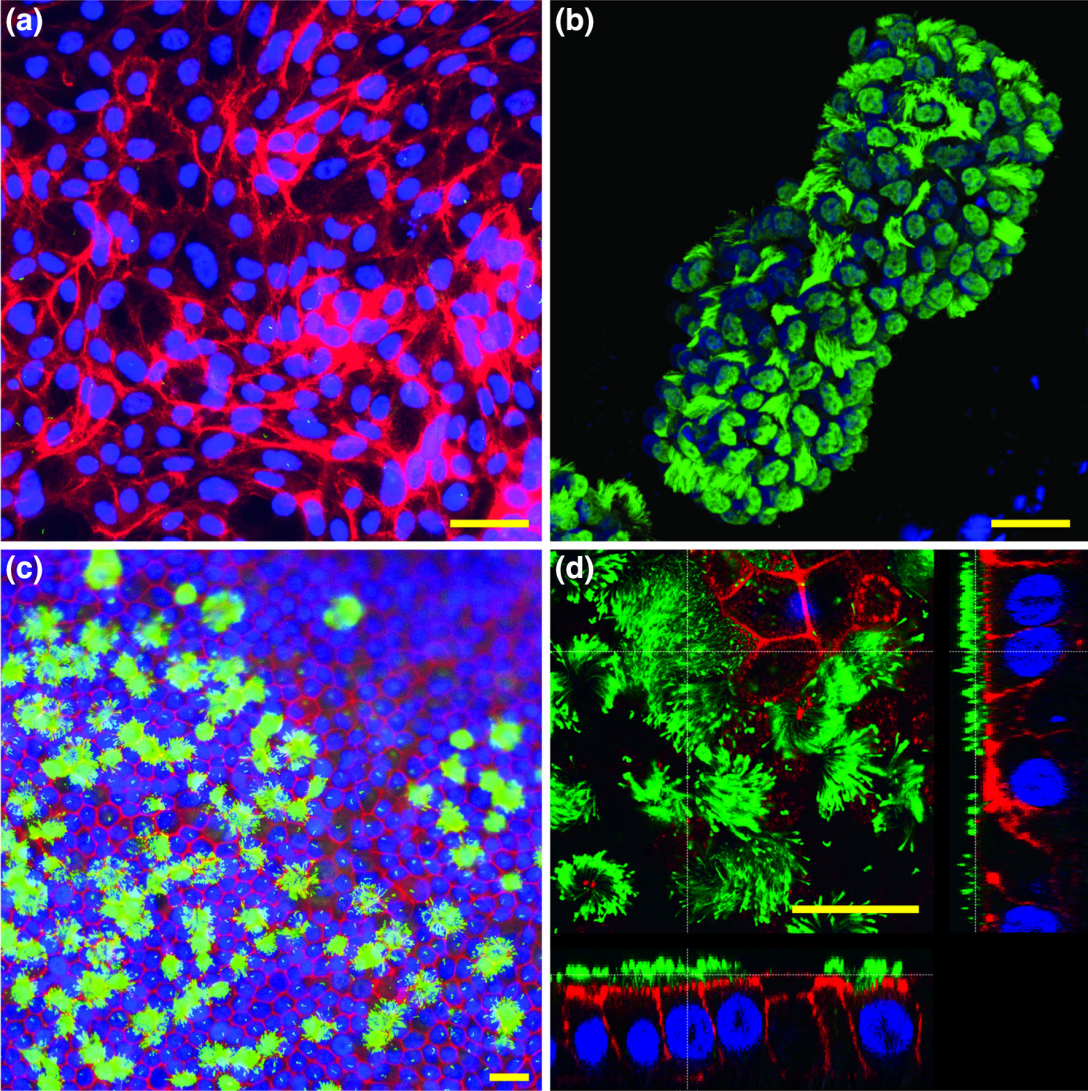
Fluorescent staining for nuclei (blue, Hoechst33342), actin filaments (red, phalloidin) and cilia (green, indirect immunofluorescent labeling of acetylated alpha tubulin) in different *in vitro* oviduct epithelial cell (OEC) culture techniques. (a) An equine 2-D OEC culture without secondary cilia. (b) A bovine OEC explant vesicle showing ciliated and non-ciliated cells (note; we did not stain this specimen for phalloidin as confluent contact between the cells in this epithelial vesicle is known to remain intact). (c) and (d) Equine OECs cultured on porous membranes for 6 weeks at an air-liquid interface; note the presence of ciliated and non-ciliated cells in C and D, and the columnar shape of the cells with nuclei at the base and cilia at the apical aspect of the cells in D. Equine images were provided by H.H.W.H (data unpublished) and bovine image by M.A.M.M.F. (data unpublished). Bars = 25* µ*m.

The most commonly reported method for investigating oviduct function *in vitro* oviduct is the monolayer culture (2-D culture; Fig. 4a). 2-D culture of OECs is hampered by a rapid loss of typical differentiated OEC properties, such as ciliation, columnar cell morphology, cell polarity, secretory granules and bulbous protrusions.^8^,^39^,^40^,^47^,^48^,^101^,^104^,^115^ The use of 2-D culture was nevertheless a useful first step in trying to understand the roles of the oviduct during gamete interaction and early embryo development. Even though OEC morphology is not preserved during 2-D culture, several studies demonstrated interactions between the OECs and spermatozoa, indicating that OECs and/or their secretions could influence sperm function.^29^,^30^,^81^,^112^ Additionally, there is evidence of beneficial effects of OECs in 2-D culture on the early embryo *via* OEC-derived embryotrophic growth factors,^114^ a decreased oxygen tension and avoidance of the block to embryonic genome activation.^28^,^37^,^84^

Another* in vitro* model used to study gamete interaction and embryo development is the oviduct explants (Fig. 4b). Oviduct explants are aggregates of epithelial and stromal cells that organize to form motile, everted vesicles with active cilia on the outer surface.^40^,^66^,^86^ OECs in oviduct explants are able to maintain their differentiated morphology as characterized by secondary cilia, numerous mitochondria and rough endoplasmic reticulum,^86^ and expression of oviductal epithelial cell markers such as oviductal glycoprotein 1 (OVGP1), glutathione peroxidase 4 (GPX4) and forkhead box protein 1 (FOXJ1).^71^ One drawback at least for bovine material is the limited viability of the explants that, within hours to days, lose their differentiated state with the epithelial cells becoming flat and non-ciliated which appears not to be such a problem for equine material.^66^,^86^ Another disadvantage is the fact that the system doesn’t mimick the air–liquid interface as it happens *in vivo* needing a large volume of medium during culture, therefore not mimicking oviductal conditions properly.

Three-dimensional culture using inserts with porous membranes and air–liquid interfaces (Figs. 3c and 3d) have been developed in recent years. This technique allows cultured OECs to retain their polarized columnar epithelial cell characteristics, and has been applied successfully to OECs from various mammalian species.^14^,^16^,^34^,^40^,^92^–^94^ Within the inserts, seeded OECs first form a confluent layer on the porous membrane. Subsequently, the medium from the apical aspect is removed to establish an air–liquid interface. As a result, the cells receive metabolites only from the basal surface, a trick that induces apical-basolateral polarity. Moreover, the OECs start to re-differentiate and begin to express secondary cilia on their apical surface from 2-3 weeks post-confluence and are able to maintain the polarized state during long term culture (for at least 6 more weeks). The resulting polarized OECs are able to bind introduced sperm^13^,^40^,^92^ and secrete factors into medium film of the insert that triggers the release of previously bound sperm.^40^ Moreover, the OECs are responsive to endocrine stimulation, as demonstrated by an increase in the expression of prostaglandin receptor (PGR), estrogen receptor 1 (ESR1) and epithelial markers such as mucin 16 (MUC16), OVGP1 and heat shock protein 90 beta member 1 (HSB90B1), when exposed to estrogens, and a decrease in the same markers when stimulated by progesterone.^13^ Despite all the potential advantages of 3-D OEC cultures, current well inserts do not permit live imaging or perfusion studies. Moreover, theses 3-D OEC systems lack the tubular folded architecture of the oviduct. These shortcomings are likely to limit their use to study gamete interactions and early embryo development in any detail.

### Organoid Models

Two different methods for developing oviduct organoids have been described^49^,^54^,^55^ and, in both, it was possible to maintain differentiated OECs within a folded tubular structure reminiscent of the *in vivo* oviduct: (1) In the first method, small pieces of oviduct were cultured inside an alginate matrix. These organoids were maintained in culture for 7 days and expressed normal oviductal epithelial cell markers, such as OVGP1, paired box 8 (PAX8), E-cadherin and cytokeratin; they also preserved a columnar epithelium with a mix of ciliated and non-ciliated cells.^54^ (2) The second method for organoid culture was based on the existence of adult stem cells in the distal part of the oviduct.^49^,^88^,^89^,^120^ Kessler and collaborators^49^ isolated these adult stem cells and cultured them in a Matrigel matrix supplemented with a cocktail of growth factors, including epidermal growth factor (EGF), fibroblast growth factor 10 (FGF-10) and transforming growth factor beta (TGF-β). The cells were able to proliferate and form spheroids, with folds appearing during the second week of culture. The resulting organoids also presented highly polarized columnar epithelial cells orientated with the apical side into the sphere’s lumen. The mature organoids presented both PAX-8 positive secretory cells and PAX-8 negative, but acetylated tubulin positive, ciliated cells and were able to maintain this morphology during long term culture (up to 8 months). Demonstrating a fully differentiated epithelium, with both ciliated and secretory cells, that can communicate directly (by the interaction between the sperm cell and the cilia, the sperm reservoir) or indirectly (by secreting factors into its lumen) with gametes and embryos. Although organoid culture can preserve oviduct morphology and OEC polarization, it has limitations in that the luminal compartment of the organoid is only accessible for gametes or embryos *via* micro-puncturing. Thus, expensive micromanipulators are required and technical expertise must be developed to further study gamete activation, fertilization and embryo development.

### Microfluidics

Research into microfluidics and reproductive events have increased in the past years, and relatively new papers on microfluidics and gamete development have been published.^4^,^15^,^20^,^21^,^24^,^31^,^41^,^42^,^52^,^56^,^62^,^78^,^99^,^111^,^121^,^124^ In most cases, these papers relate to sperm migration, and none have included OECs in the model. Interestingly, microfluidics devices have been designed to study sperm rheotaxis, movement, thermotaxis and chemotaxis, thereby mimicking physical and chemical factors that sperm encounter during their passage through the female tract (for a detailed description see Suarez and Wu^109^). Zhang and collaborators^124^ included oviductal fluid to help select sperm cells *via* a microfluidics system. Using this combination, they were able to observe sperm migration and select sperm with better motility and DNA integrity, concluding that it was a useful tool for selecting sperm for IVF procedures. It was also demonstrated that sperm rely more on the channel geometry than chemotaxis (*i.e.* sperm cells preferentially swim along boundaries and, when two boundaries intersect, the cells will follow the corner, swimming along one-dimensional folds^24^). Although the folding of the oviduct is more complex than the walls of a fabricated microchannel, these results suggest that the 3-D architecture of the compartment in which sperm migrate is important and that the topography of the oviduct wall may help guide the spermatozoa to the oocyte *in vivo*.^24^

Angione and collaborators^4^ engineered a microfluidic device that allows precise and flexible handling of individual oocytes and embryos. Their system allowed perfusion and live imaging of the introduced oocytes or developing embryos that could be used for both clinical and research IVF purposes. Nevertheless, most current embryo culture systems are static,^111^ although interest in microfluidic devices for embryo culture systems has increased in recent years. Potential benefits of a dynamic (microfluidic) embryo culture system are continuous removal of harmful products and replenishment of substrates, disruption of unwanted environmental gradients, physical stimulation and activation of signaling pathways.^111^ Mechanical stimulation of bovine embryos in a microfluidic device increased the proportion of 2-cell embryos developing into 8-cell embryos, when a constrictive channel was used (increasing from 23.9 to 56.7%).^52^ Mechanical shear stresses imposed should not however be too harsh because embryos degenerate at values above 1.2dyn/cm.^2^ ^121^ A “womb-on-a-chip” was designed to establish a dynamic co-culture between endometrial cells and the embryo.^78^ This system allows investigation of the interaction between the embryo and secretions from the endometrial cells, moreover the co-culture resulted in improved murine blastocyst rates.^78^ Nevertheless, a similar approach using microfluidics combined with OECs to enhance embryo production has not yet been reported.

### 3-D Printing

Micro-engineered 3-D cell cultures, in which cells are maintained in micro-3-D fabricated devices that mimic tissue- and organ-specific micro-architecture,^43^ have recently attracted attention. These approaches promote levels of cell differentiation and polarization that are not readily achieved by normal 2-D cultures. Nowadays, 3-D printing offers a fast prototyping process technology, such that researchers can design and print devices in a short period of time.^69^ Combined with microfluidics, these techniques can lead to rapid creation and refinement of organs-on-a-chip to study human and animal organ-specific physiology and may, thereby, offer better *in vitro* organ models for research into aspects of physiology, disease and toxicology.^43^

**Figure 5:**
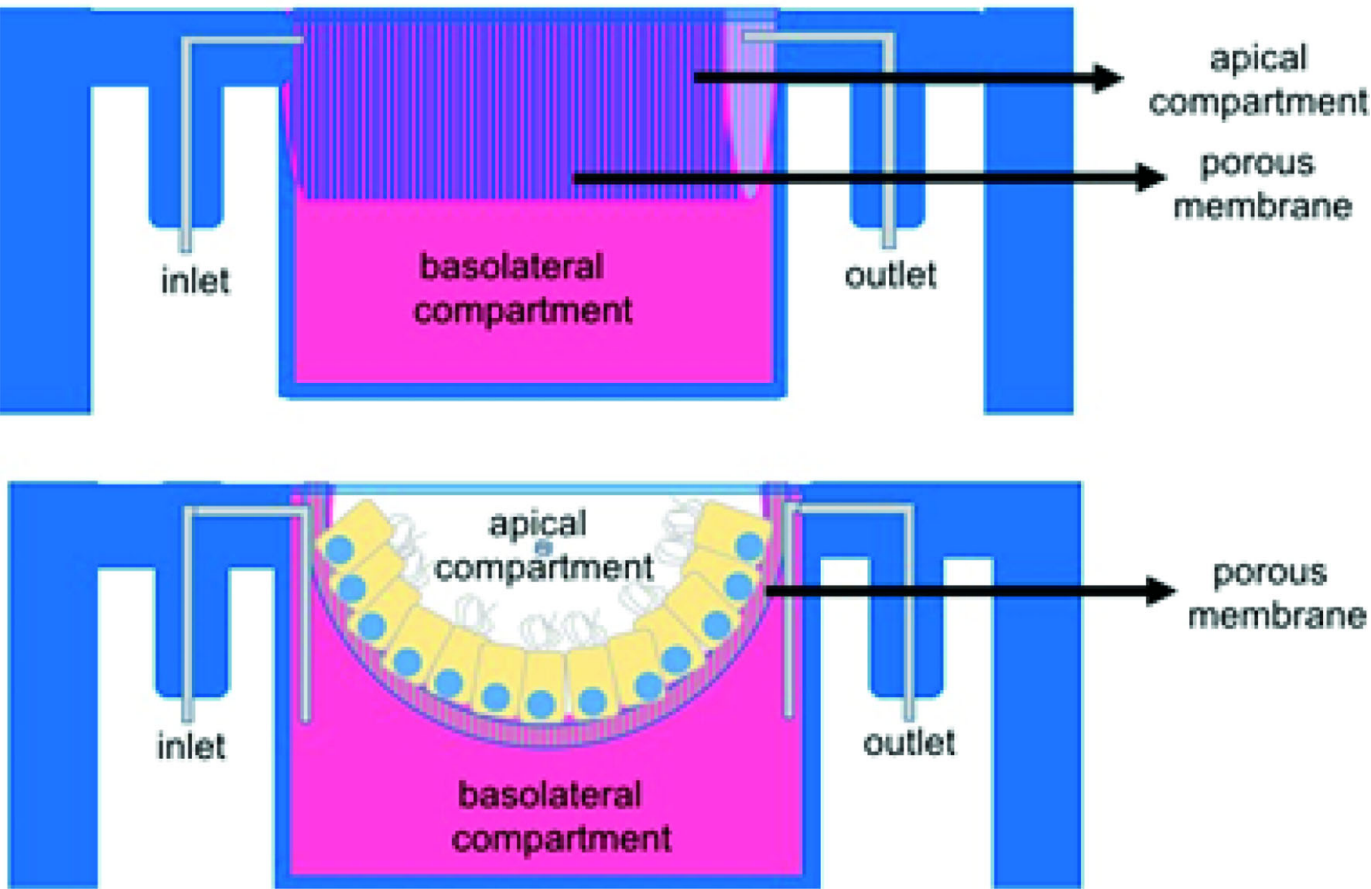
The 3-D printed oviduct-on-a-chip cultures. Also in this culture systems two compartments are formed (apical and basolateral) that are separated by a porous membrane and a confluent layer of oviduct epithelial cells. Note the inlets and outlets for independent perfusion of the apical and basolateral compartments, and the folded U-shape structure, that are introduced into the 3-D printed oviduct-on-a-chip.

3-D printing has been used to fabricate various tissues including bone, cartilage, skin, heart tissue, and vascular tubes.^82^ To our surprise, we were the first to use 3-D printing technology in combination with microfluidics for assisted reproduction, when developing an *oviduct*-*on*-*a*-*chip* model.^72^ We designed and 3-D printed, using the stereolithography technique, a tubular like insert in which OECs could be cultured at an air–liquid interface and acquire and maintain epithelial polarization and differentiated cell state during long-term culture. The 3-D culture and polarization of OECs in our 3-D printed inserts resembles that of the cell insert approach with porous membranes (Fig. 3). However with the new 3-D OEC system, live imaging is possible, sperm can bind to the apical side of the OEC and be released. Furthermore, the system promotes normal fertilization and is easy to manipulate (*i.e.* for adding or removing gametes, embryos and cell secretions). The system also allows independent double perfusion (*i.e.* of the apical and basolateral medium compartments independently; Fig. 5) while maintaining a tubular morphology that could be made more complex to better mimic the oviduct. Furthermore the cells can keep a polarized state for long term cultures (at least six weeks), without loosing ciliation and ability to promote sperm activation (Ferraz et al., unpublished results).

Therefore, the *oviduct*-*on*-*a*-*chip* is a step forward for mimicking the interaction between gametes and embryos and the maternal oviductal environment. This will yield a better and more accessible bio-mimicking tool to study oviduct physiology and improve understanding of reproductive health and disease, as well as for screening toxicological compounds and novel drugs.

## Improvements in Oviduct Modelling *Via* Bioengineering

Better 3-D cell culture systems to bio-mimic the oviduct can help to improve our understanding of *in vivo* processes that take place in this organ, and should help to improve the efficacy of assisted reproductive technologies (ARTs). The oviduct has an essential function in guiding and regulating sperm activation, oocyte maturation, fertilization and early embryo development.^28^,^38^,^51^,^68^,^91^,^114^ A better understanding of how the oviduct orchestrates these processes could aid in the development of better sperm storage and cryopreservation techniques.^25^,^105^ Moreover, improved oocyte maturation and IVP results, including a reduction in polyspermic fertilization and parthenogenetic activation, can also be achieved.^17^,^18^,^45^,^68^,^80^ Another aspect of improved IVP embryo quality could be survival after cryopreservation, reduced lipid content and avoidance of epigenetic changes that can impair embryo development or offspring health.^5^,^7^,^9^,^22^,^27^,^46^,^67^,^87^,^97^,^98^,^100^,^113^ Conventional *in vitro* fertilization (IVF) and embryo production has species specific problems. For instance, equine oocytes cannot be fertilized *in vitro* unless intracytoplasmic sperm injection (ICSI) is used, which requires expensive and dedicated technology and is labour intensive.^3^,^65^,^102^ For cattle, it is known that *IVP* embryos are of lower quality and have reduced cryosurvival compared to embryos flushed from the uterus.^85^,^110^,^119^ Both examples, clearly indicate that the oviductal environment is more conducive to producing good quality embryos than any *in vitro* system tested to date. Future studies will reveal whether or not the oviduct-on-a-chip approach will offer a superior oviduct-like environment for improved embryo production. A working oviduct-on-a-chip system would also offer a novel approach to reproductive toxicology testing or pharmaceutical agent screening, and for male and female infertility testing.

As we move from 2-D cultures to micro-engineered organs-on-a-chip, new challenges will undoubtedly arise. For instance, optimizing biological (cell) and non-biological (materials) culture requirements, optimizing/allowing cell polarization, differentiation and preventing possible toxic effects of the materials used. A multidisciplinary approach will be necessary to solve the likely challenges and maximally exploit the new opportunities the organ-on-a-chip technique will offer. In the more distant future, more complex bioengineered tissues (such as multilayered oviduct, follicles and endometrial cell cultures) could be combined to create a female-reproductive-tract-on-a-chip. However, at present we believe that the oviduct-on-a-chip technology is closer to being ready, and has more obvious immediate applications in the field of ART.

## Abbreviations

2-D: Two-dimensional
3-D: Three-dimensional
ARTs: Assisted reproductive techniques
BM: Basal membrane
COC: Cumulus oocyte complex
EGF: Endothelial growth factor
ESR1: Estrogen receptor 1
FCM: Fluorescent confocal microscopy
FGF-10: Fibroblast growth factor 10
FOXJ1: Forkhead box protein 1
GJ: Gap junctions
GPX4: Glutathione peroxidase 4
HSB90B1: Heat shock protein 90 beta member 1
ICSI: Intracytoplasmic sperm injection
IVP: *In vitro* embryo production
MUC16: Mucin 16
OEC: Oviduct epithelial cells
OVGP1: Oviductal glycoprotein 1
PAX8: Paired box 8
PGR: Progesterone receptor
TGF-β: Transforming growth factor beta
